# Dairy Effluent-Saturated Biochar’s Short-Term Effects on *Vigna unguiculata* and *Cynodon dactylon* Performance and Soil Properties

**DOI:** 10.3390/plants13060851

**Published:** 2024-03-15

**Authors:** Lisandro J. Entio, Cosette B. Taggart, James P. Muir, Eunsung Kan, Jeff A. Brady, Olabiyi Obayomi

**Affiliations:** 1 Texas A&M AgriLife Research and Extension Center at Stephenville, 1229 North US Highway 281, Stephenville, TX 76401, USA; cosette.taggart@go.tarleton.edu (C.B.T.); eunsung.kan@ag.tamu.edu (E.K.); jeff.brady@ag.tamu.edu (J.A.B.); biyi.obayomi@ag.tamu.edu (O.O.); 2 Wildlife and Natural Resources Department, Tarleton State University, Stephenville, TX 76401, USA

**Keywords:** effluent-saturated biochar, soil characteristics, *Vigna unguiculata*, *Cynodon dactylon*

## Abstract

We compared the effects of wood-, manure-, and blend-derived biochar (BC) saturated/unsaturated with dairy effluents on *Vigna unguiculata* and *Cynodon dactylon* performance and soil characteristics in a greenhouse pot study. Plant samples were assayed for herbage and root dry weight and N and C percentages. Soil samples were assayed for nutrients, pH, and conductivity. Variance analysis, Tukey’s tests, Pearson’s correlations, and multiple regression analysis were performed. The performance of *C. dactylon* was not affected. *V. unguiculata*’s herbage and root production responded negatively to manure BC and 2% of any BC, respectively, which is mainly explained by the conductivity and soil P increase, respectively. When *V. unguiculata* was grown, BC inclusion decreased NO_3_-N and increased the soil P content. When *C. dactylon* was grown, only P was altered (increased) when manure or the blend BC were applied. The soil total C increased as the BC loading rate increased. The application of high BC rates was detrimental for *V. unguiculata,* but showed a neutral effect for *C. dactylon*. To improve dairy waste recycling, saturated 1% blend BC and saturated 2% blend or manure BC could be applied to *V. unguiculata* and *C. dactylon*, respectively, with no short-term negative impacts. Only wood BC avoided soil P build-up. BC application increased the soil total C, showing potential for C sequestration.

## 1. Introduction

Global agricultural activities account for approximately 30% of total greenhouse gas emissions, mainly due to the use of chemical fertilizers, pesticides, and animal waste [1]. Carbon sequestration (the capture and long-term storage in the soil of atmospheric carbon dioxide (CO_2_)) currently represents the best solution to counter the rise in greenhouse gases [1]. In the last two decades, and based on early studies already conducted by the end of the 19th century on “Terra Preta” soils in the Amazon, the use of biochar (BC) began to catch the attention of the scientific community as a strategy for carbon storage for climate change mitigation. Several authors reported that BC not only contributes to soil C sequestration, but also the widespread use of BC as a soil amendment can improve a broad range of soil properties, such as water- and nutrient-holding capacity, thereby enhancing agricultural yields [2,3,4]. Moreover, Biederman and Harpole (2012) [5] highlighted BC as a beneficial solution for energy production. 

BCs are rich stable organic C products obtained by plant biomass pyrolysis generally at temperatures between 300 and 700 °C under limited oxygen conditions. They are typically derived from agricultural and forestry waste products, municipal waste, and green waste or food waste [6]. BC can be produced as a biproduct of biofuel production [7,8] or as the desired end product of pyrolysis. BC’s large surface area and greater negative surface charge and charge density [9] than soil organic matter make it more efficient in terms of cation adsorption per unit carbon [10]. It can also adsorb nutrients and organic matter from soils and nutrients from added manure or fertilizer [11], as well as from anaerobic digestates [12] and livestock wastewater [13], thereby mitigating environmental nutrient pollution [7]. BC can contribute to a more circular nutrient cycling at farm and regional scales by improving nutrient recovery and nutrient use efficiency [14].

Nowadays, the use of cow manure as a soil amendment and plant nutrient supply is a worldwide practice [15]. The 9.38 million dairy cows in the United States of America alone [16] produce up to 13.5 Mg/year of manure [17] that, when applied to soil as an amendment (i.e., solid or liquid/slurry amendment), generally based on N-targeted crop needs, or when discarded in non-agricultural use lands, may lead to P [18] and pathogen [19] oversaturation. This increases the probability of surface runoff with consequences like eutrophication and/or water contamination. However, pyrolyzed cow manure (i.e., derived manure BC) could be a suitable option for dairy waste recycling/reuse to create a tighter loop between inputs and outputs that minimizes externalities and improves soil carbon storage. The use of BC to capture the excess nutrients or pathogens in common agricultural pollutants, such as dairy wastewater, can offer an economical solution for disposing of excess biomass while improving the soil nutrient content, holding capacity, and carbon sequestration [20].

Several studies have documented BC’s potential in improving the productivity of crops and soil characteristics. However, whether BC has a positive, negative, or neutral impact on plant performance and soil parameters, especially in the short term, is poorly documented in forage systems. The most suitable biochar type and application rates are still unknown for most forage production systems (i.e., soil type, species, etc.), especially in the case of dairy effluent-enriched/saturated BC application. For instance, the addition of enriched BC can improve soil quality (i.e., organic matter and macronutrients) and the biomass yield of maize [12]. Enriched BC increases soil nutrients (e.g., N and P) and can result in greater *C. dactylon* biomass in the short term [21]. Likewise, positive short-term effects on *Lolium multiflorum* growth occur when different types of saturated BCs are applied, while the same BC (enriched or not) has a negative effect on *Trifolium incarnatum* performance, as well as varied effects on soil parameters [22]. For the performance of *Vigna unguiculata,* BC can have a neutral effect [23], while under other conditions, a positive effect [24,25,26,27] occurs, as well as varied effects on soil parameters. 

A better understanding of BC amendments’ (i.e., type, nutrient saturation, and rate) short-term effects in combination with dairy effluent saturation and their impacts on plant and soil properties will be useful not only for decisions about the use of manure effluent and BC to enhance warm-season pastures (annual and perennial) and soil properties, but also about dairy waste recycle/reuse possibilities for tighter input–output options at the farm and regional scales for agricultural production. 

Although several studies have determined the effects of BC on plant growth and soil fertility, few have examined the effects of BCs made from different feedstocks saturated or not with dairy effluent nutrients on forage growth and soil parameters. In our greenhouse pot study, the objective was to compare the short-term effects of unsaturated (raw) BCs [wood, manure, and blend (50% wood/50% manure)] and dairy effluent nutrient-saturated BCs on plant performance (i.e., production and nutritive values of *V. unguiculata* and *C. dactylon*) and soil characteristics. These annual forage species were chosen because of their adaptation to low rainfall and warm-season temperatures in sub-tropical regions known to have soils deficient in the key nutrients and micronutrients required by grass and legume forages. 

## 2. Results

### 2.1. Plant Performance

The first *V. unguiculata* herbage cut (g H-DW1/pot) was affected by the “BC type × BC loading rate” interaction. Pots containing 2% manure BC differed from both the control and the other treatment combinations, showing 61% less dry matter than control. With respect to H-DW1 quality, N% was not affected, whereas C% was affected by the two-factor interaction “BC type × BC loading rate”. In this manner, only 2% manure BC affected herbage C content by reducing it by 8% (Table 1).

The first *C. dactylon* herbage cut (g H-DW1/pot) was not affected by any treatment, nor a combination of them. With respect to H-DW1 nutritive value, N% was not affected, whereas C% was affected by the main factor “BC loading rate”. In this manner, only 2% BC load decreased C% content compared with control (Table 1). 

The second *V. unguiculata* herbage cut (g H-DW2/pot) yield was affected by the two-factor interactions “BC type × BC loading rate” and “BC type × BC saturation”. For the “BC type × BC loading rate” interaction, only manure BC, regardless of the loading rate, affected dry matter by reducing it by 40% (Table 2). With respect to “BC type × BC saturation,” there was no difference between saturation treatment for any BC type; however, when saturated, blend BC resulted in greater herbage yields than manure BC but equal to wood BC. Wood BC showed no difference with manure BC. H-DW2 nutritive values were not affected by any treatment, nor a combination of them (Table 2). 

The second *C. dactylon* herbage cut (g H-DW2/pot) was affected by the main factor “BC loading rate”. The 1% BC loading rate reduced herbage yields by 27% compared with the control, although it showed no differences with the 2% BC loading rate treatment. The H-DW2 nutritive value was not affected by any treatment, nor a combination of them (Table 2).

The *V. unguiculata* total herbage dry weight (g TH-DW/pot) was affected by a “BC type × BC loading rate” interaction. The 1 and 2% manure BC differed with their respective controls, reducing TH-DW/pot by 18% and 70%, respectively (Table 3). 

The *C. dactylon* total herbage dry weight (g TH-DW/pot) was not affected by any treatment, nor a combination of them.

The *V. unguiculata* root dry weight (g R-DW/pot) was only affected by the main factor “BC loading rate”. The 2% BC loading reduced it by 40% compared with the control, although it showed no differences with the 1% BC loading treatment (Table 4). However, R-DW quality was not affected by any treatment, nor a combination of them.

With respect to *C. dactylon* root dry weight (R-DW), neither herbage yield nor nutritive values were affected by any treatment, nor a combination of them. 

### 2.2. Soil Parameters

#### 2.2.1. Macronutrients

Total soil C in *V. unguiculata* was affected by the main factors “BC type” and “BC loading rate”. Thus, the incorporation of wood and manure BC showed the greatest and lowest values, respectively, showing no differences with the application of blend BC. Regarding “BC loading rate,” total C values increased as BC application rate increased (2% BC > 1% BC > 0% BC) (Table 5).

Oxidizable soil C was affected only by the main factor “BC saturation”. Saturated BC treatment showed 43% more C compared with the unsaturated treatment (Table 5); however, saturated BC treatments showed a similar value to control pots with no amendments or plant application, whereas unsaturated BC showed 29% less oxidizable C than control.

Total N was not affected by any treatment or a combination of treatments, whereas bio-available NO_3_-N was affected by the main factors “plant application” and “BC saturation”. The inclusion of *V. unguiculata* reduced soil N content by 88% compared with bare soil and the application of saturated BC reduced it by 35% when compared with the unsaturated BC (Table 5). The unsaturated and saturated BC treatments showed a tendency (*p* > 0.05) to have less (32 and 56%, respectively) bio-available NO_3_-N than the control pots with no amendment or plant application.

P was affected by the main factor “BC saturation” and the three-factor interaction “BC type × BC loading rate × plant application”. Soil containing saturated BC treatment showed 18% less P compared with the unsaturated treatment (Table 5); however, both treatments showed a tendency (*p* > 0.05) to exceed the P content of the control pots (35 mg/kg). With respect to the three-factor interaction “BC type × BC loading rate × plant application,” the incorporation of manure or blend BC at the lowest loading (1%) had similar effects, increasing soil P by an average of 173% compared to controls regardless of whether a plant was present or not. No differences (*p* > 0.05) were observed for these treatment combinations when compared with the highest loading (2%), except for blend BC when *V. unguiculata* was present (2% blend BC + plant > 1% blend BC + plant). Regarding wood BC treatments, there was no effect between any treatment combination, nor between treatments and controls (Table 6).

Like P, K was affected by the main factor “BC saturation” and the three-factor interaction “BC type × BC loading rate × plant application”. Saturated BC treatment showed 12% less K compared with the unsaturated treatment (Table 5); however, both treatments showed a tendency to exceed the bio-available K content of the control pots (199 mg/kg). With respect to the three-factor interaction, regardless of whether a plant was present or not, 2% manure BC showed the highest soil K content, averaging 398%. However, both manure and blend BC increased K level even at the lowest BC loading rate by an average of 118.5% (Table 6).

Na was affected by the main factor “plant application” and the three-factor interaction “BC type × BC loading rate × BC saturation”. The inclusion of *V. unguiculata* resulted in 39% more Na compared with bare soil treatment (Table 5). With respect to the three-factor interaction, regardless of whether BC was saturated or not, manure or blend BC treatments at the highest loading rate (2%) had similar effects, increasing Na an average of 111% compared to the control. Unsaturated manure BC increased Na even at the lowest loading rate (1%). Regarding wood BC treatments, there was no effect between any treatment combination, nor between treatments and controls (Table 6). 

Ca was affected only by the main factor “BC type”. The application of manure BC showed 5% more Ca than blend or wood BC, which did not differ from one another (Table 5). However, the control soil Ca content was 1658 mg/kg.

Mg was affected by the main factor “plant application” and the two-factor interaction “BC type × BC loading rate”. The inclusion of *V. unguiculata* showed 16.5% more Mg compared with the bare soil treatment (Table 5). With respect to the two-factor interaction, 2% manure BC showed the highest value, increasing soil Mg content by 87% compared to the control. Even at the lowest loading rate (1%), manure BC exceeded the control by 52%. Moreover, within each loading treatment, manure BC resulted in a higher soil Mg content than blend and wood BC. Blend biochar had a higher Mg concentration than wood biochar and increased soil Mg content (47%) at the highest loading rate. Wood BC did not differ from the control at any loading rate (Table 6).

S was affected by the two-factor interactions “BC type × BC loading rate” and “BC saturation × plant application”. Regarding the “BC type × BC loading rate” interaction, only manure BC application showed differences compared to the control, increasing S soil content 38% (0 < 1% = 2%) (Table 6). With respect to “BC saturation × plant application” interaction, when unsaturated BC was applied, plant presence reduced soil S content by 19%; however, when saturated BC was applied, plant presence had no effect. In any case, S values were higher than or similar to the control pots’ S content (24.33 mg/kg) (Table 6). 

Regarding *C. dactylon*, combustible total C was affected by the main factor “BC loading rate”. Thus, total soil C values increased from 2.81% (0%BC) to 3.98% (2%BC) as the application rate increased (2% > 1% > 0%) (Table 5). Oxidizable C was not affected by any main factor or interaction.

Total soil N content was not affected by any treatment or combination, whereas NO_3_-N was affected by the main factor “plant application”. The inclusion of *C. dactylon* reduced this nutrient by 86% compared with bare soil (Table 5).

P was affected by the main factor “plant application” and the two-factor interaction “BC type × BC loading rate”. The inclusion of *C. dactylon* reduced it by 18% compared with bare soil (Table 5). With respect to the “BC type × BC loading rate” interaction, manure BC increased the soil P content at the lowest and highest loading rates (183 and 314%, respectively), while blend BC showed no statistical differences between loading rates, increasing the soil P content by 135%. When applied at the lowest loading rate, manure and blend BC showed the same effect, whereas, at the highest loading rate, manure BC had a greater effect. Wood BC treatment did not differ from the control (Table 6).

K was affected by two-factor interactions “BC type × BC loading rate” and “BC loading rate × plant application”. For “BC type × BC loading rate” interaction, manure or blend BC increased K soil content as their loading rate increased; however, the manure BC showed higher values than the blend BC at both loading rates. The application of wood BC did not differ from the control at any loading rate (Table 6). With respect to the “BC loading rate × Plant application” interaction, within each BC loading rate, the inclusion of *C. dactylon* showed a similar soil K reduction (around 20%) when compared with bare soil. Regardless of whether a plant was present or not, soil K content increased by at least 66% as BC loading rate increased (Table 6). 

Na was affected by the three-factor interaction “BC type × BC loading rate × BC saturation”. Unsaturated manure BC and unsaturated blend BC at the highest loading (2%) had similar effects, increasing soil Na by an average of 153% compared to control. Only unsaturated manure BC increased bio-available Na soil content. The 1% unsaturated manure BC loading rate showed no differences with the 2% loading rate. Neither wood BC nor saturated blend BC treatments showed differences with the control (Table 6).

Ca was affected by the main factors “plant application” and “BC type”. The inclusion of *C. dactylon* reduced soil Ca by 7% compared with bare soil. The application of the manure BC resulted in 8% more Ca than did the wood BC application, whereas the blend BC did not differ from either of them (Table 5). The soil Ca content in the control pots was 1658 mg/kg.

#### 2.2.2. Micronutrients 

Mn for *V. unguiculata* was affected only by the main factor “BC type”. Wood BC showed on average 16% more Mn than the manure BC or the blend BC, which were roughly equal to one another (Table 7). 

Cu was affected by the main factor “plant application” and the two-factor interaction “BC type × BC saturation”. The inclusion of *V. unguiculata* resulted in 23% more Cu than bare soil treatment (Table 7). With respect to the two-factor interaction, only saturated blend BC differed (−24%) from its unsaturated treatment (Table 8). The Cu content in control pots was 0.33 mg/kg.

Fe was affected by the main factors “plant application” and “BC loading” and by the two-factor interaction “BC type × BC saturation”. The inclusion of *V. unguiculata* showed 13% more Fe than the bare soil treatment, and the addition of BC, regardless the loading rate, decreased Fe soil content by an average of 23% (Table 7). With respect to the two-factor interaction “BC type × BC saturation,” similar to Cu, only saturated blend BC differed (−24%) from its unsaturated treatment in Fe content (Table 8). The Fe control pot content was 3.52 mg/kg.

Zn was affected by the main factors “plant application” and “BC saturation”. The inclusion of *V. unguiculata* reduced Zn by 8% compared with bare soil, and the application of saturated BC was 7% less when compared with unsaturated BC (Table 7).

Regarding *C. dactylon*, Mn was affected by the main factor “plant application” and the two-factor interactions “BC type × BC loading rate” and “BC type × BC saturation”. The inclusion of *C. dactylon* reduced it by 10% compared with the bare soil treatment (Table 7). For the “BC type × BC loading rate” interaction, only the 2% wood BC treatment increased (31%) the soil Mn content. The manure or blend BC treatments showed no differences with the control treatment. With respect to “BC type × BC saturation” interaction, only the application of saturated blend BC showed a lower value when compared with its respective unsaturated treatment (Table 8).

Cu was affected by the two-factor interaction “BC type × BC saturation”. Only the saturated blend BC differed (−20%) from the unsaturated treatment (Table 8). The Cu control pot’s average content was 0.338 mg/kg.

Unlike Zn, which was not affected by any main factor or interaction, Fe was affected by the main factors “plant application” and “BC loading rate” and by the two-factor interaction “BC type × BC saturation”. The inclusion of *C. dactylon* showed 23% more Fe than the bare soil treatment, and the addition of BC, regardless of the loading rate, decreased Fe soil content by an average of 27% (Table 7). With respect to the two-factor interaction “BC type × BC saturation,” similar to Cu, only the saturated blend BC differed (−18%) from the unsaturated treatment (Table 8). The Fe control soil content was 3.54 mg/kg.

#### 2.2.3. pH and Conductivity

For *V. unguiculata*, soil pH was affected by the two-factor interactions “BC type × BC loading rate,” “BC type × plant application,” and “BC type × BC saturation”. For the “BC type × BC loading rate” interaction, the manure and blend BC types increased pH as the loading rate increased, and the wood BC increased it only at the highest loading rate. At the lowest BC loading rate, the manure BC effect was similar to the blend BC, but superior to the wood BC, whereas the blend and wood BC showed no differences. At the highest BC loading rate, all treatments increased pH [manure BC (9%) > blend BC (5%) > wood BC (2.5%)] (Table 9). Regarding “BC type × plant application” interaction, the presence of *V. unguiculata* differentially reduced soil pH only in manure and wood BC treatments (0.63 and 3%, respectively) (Table 9). With respect to “BC type × BC saturation”, there was no difference between saturation treatments for any BC types; however, when saturated, the manure BC showed the highest value (Table 9).

Conductivity was affected by the two-factor interactions “BC type × BC loading rate” and “BC loading rate × plant application”. For the “BC type × BC loading rate” interaction, only manure BC treatment differed from the control by increasing conductivity as BC loading increased (2% > 1%) (Table 9). With respect to the “BC loading rate × Plant application” interaction, there were no differences between bare soil and *V. unguiculata* application, nor within bare soil treatment at any BC loading rates; however, within *V. unguiculata* treatment, the 2% manure BC showed the highest value, while the blend or wood BC showed no differences between them (Table 9). 

Regarding *C. dactylon*, soil pH was affected by the two-factor interactions “BC type × BC loading rate” and “BC type × BC saturation”. For the “BC type × BC loading rate” interaction, the application of manure BC affected pH both at the lowest and highest BC loading rates, increasing it by 4% and 8%, respectively, whereas application of the blend BC increased it (4%) only at the highest loading. However, at the highest loading rate, the manure BC showed a higher value than blend BC. Wood BC application showed no differences when compared to the control at any assayed loading rate (Table 9). With respect to the “BC type × BC saturation,” there was no difference between saturation treatments for any BC type; however, among unsaturated treatments, the manure BC showed the highest value (Table 9).

Conductivity was affected by the main factor “plant application” and the two-factor interaction “BC type × BC loading rate”. The inclusion of *C. dactylon* showed 10% less conductivity than the bare soil (Table 9). With respect to “BC type × BC loading” interaction, only the 2% manure BC treatment differed from the control by increasing the soil conductivity by 71% (Table 9). 

##### Pearson’s Correlations and Multiple Regression (Forward) Analysis

*Vigna unguiculata* total herbage dry weight correlated with K (r = −0.83), Na (r = 0.64), Mn (r = 0.81), S (r = 0.78), pH (r = −0.84), and conductivity (r = −0.86) when the manure BC was applied regardless of the loading rate (Table 10). Moreover, *V. unguiculata* root dry weight was correlated with several soil parameters [i.e., P (r = −0.64), K (r = −0.63), Na (r = −0.48), Mg (r = −0.49), pH (r = −0.57)] when 2% BC was applied regardless of the BC type (Table 10).

**Figure 1:**
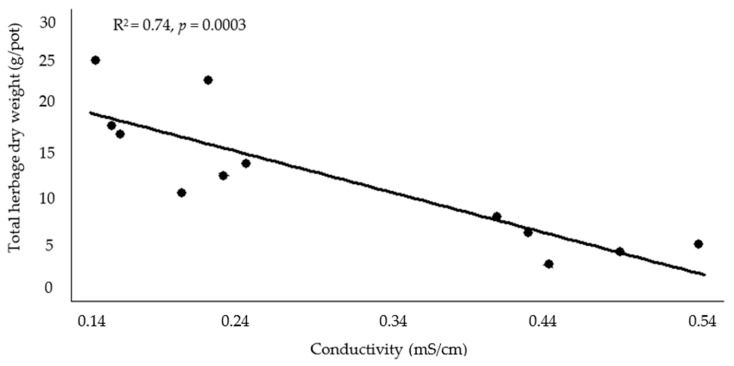
Total herbage dry weight vs. soil conductivity; multiple regression analysis (forward) for *Vigna unguiculata* when manure biochar was applied.

*Vigna unguiculata* total herbage dry weight vs. the previously mentioned correlated soil parameters (forward multiple regression analysis) showed that conductivity was the only significant variable (*p* = 0.0003) of the model and that this parameter explained 74% of the total herbage dry weight decrease in this species (Figure 1).

Conductivity was also correlated with soil parameters C (r = 0.64), P (r = 0.77), K (r = 0.88), Na (r = 0.71), Mg (r = 0.67), Mn (−0.69), S (r = 0.77), Zn (r = 0.67), and pH (r = h 0.89) (Table 10) when manure BC was applied regardless of the loading rate. 

**Figure 2:**
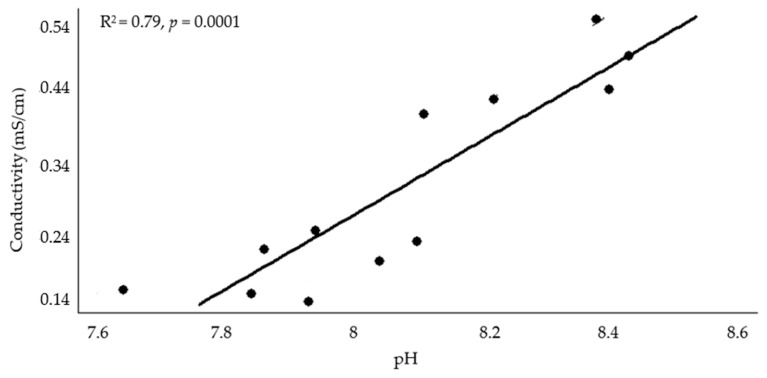
Conductivity vs. pH; multiple regression analysis (forward) for *Vigna unguiculata* when manure biochar was applied.

When comparing conductivity vs. the previously mentioned correlated soil parameters (forward multiple regression analysis), pH was the only variable (*p* = 0.0001) left in the model, explaining 79% of the conductivity increase (Figure 2).

**Figure 3:**
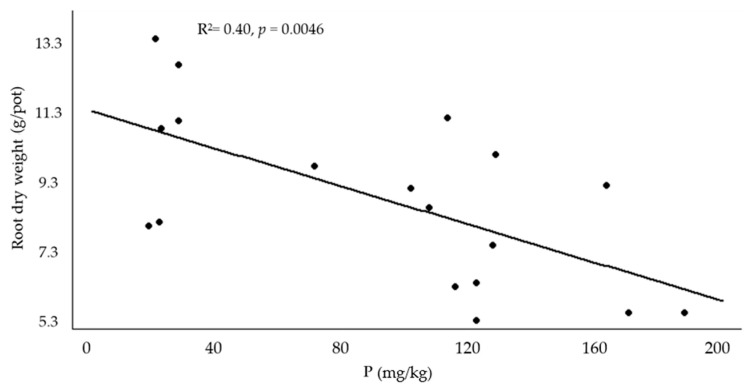
Root dry weight vs. P; multiple regression analysis (forward) for *Vigna unguiculata* when 2% biochar was applied.

Comparing *V. unguiculata* root dry weight vs. the previously mentioned soil parameters (forward multiple regression analysis) showed that P was the only variable (*p* = 0.004) in the final model, explaining 40% of this species’ root herbage dry weight decrease (Figure 3).

## 3. Materials and Methods

### 3.1. Experimental Design

This greenhouse pot study was conducted at the Texas A&M AgriLife Center in Stephenville, TX, USA (32.2454° N, −98.1970° W) over a 90-day period. Each pot was considered an experimental unit, and all treatment combinations were replicated three times. This was essentially two parallel (forage species) three-factorial experiments: (1) BC type; (2) BC effluent saturation; and (3) BC loading percentage. The plant species was not considered a factor because initial analyses of variance indicated that each species responded to the other factors in very distinct ways. Therefore, identical studies were carried out simultaneously on *V. unguiculata* and *C. dactylon* Jiggs. The study focused on these warm-season forage species because they are of widespread use within central Texas as well as throughout the semi-tropics.

### 3.2. Soil Preparation

Soil was collected from the top 20 cm of a Windthorst fine sandy loam in Stephenville, TX, USA (Table 11). It was homogenized, air-dried under ambient conditions, sifted, and distributed in 3 kg units to 162 4 L plastic nursery pots. A sandy loam was selected because it is a common texture in this region and BC amendments tend to be more effective in course-textured soils [22].

### 3.3. Biochar

Three types of BC were utilized in this study, originating from manure (Ecochar, Evansville, IN, USA), wood (Waste to Energy, Inc., South Slocomb, AL, USA), or a manure/wood blend (50% each). The physiochemical characteristics are described in Table 12. The BC was ground using a Thomas Wiley Mill (Swedesboro, NJ, USA) fitted with a 2 mm screen and used as a control (NO) or saturated (YES) in dairy manure effluent collected from the 2nd Lagoon at Tarleton State University’s Southwest Regional Dairy Center (Stephenville, TX, USA), which feeds a total mixed ration in a confined animal operation. The saturation process consisted of combining BC and dairy effluent in a 1:1 ratio to create a slurry of S^+^ BC. Slurries were homogenized every day for 14 days after which they were allowed to evaporatively dry at ambient temperatures. Once dry, the S^+^ BC was sifted to allow for proper incorporation into soils. BCs were incorporated in pots, replacing 1% or 2% of the soil in terms of dry matter weight percentage (i.e., 30 g or 60 g BC/2970 or 2940 g of soil, respectively).

### 3.4. Treatments

Each species’ experiment included three factors: BC source (manure, wood, blend), BC saturation with manure effluent (S^−^, S^+^), and BC loading percentage (0, 1, 2). Twenty-six distinct treatment combinations resulted: (1) soil (control); (2) soil + forage (control); (3, 4) 1 or 2% manure S^−^ BC; (5, 6) 1 or 2% manure S^−^ BC + forage; (7, 8) 1 or 2% manure S^+^ BC; (9, 10) 1 or 2% manure S^+^ BC + forage; (11, 12) 1 or 2% wood S^−^ BC; (13, 14) 1 or 2% wood S^−^ BC + forage; (15, 16) 1 or 2% wood S^+^ BC; (17, 18) 1 or 2% wood S^+^ BC + forage; (19, 20) 1 or 2% blend S^−^ BC; (21, 22) 1 or 2% blend S^−^ BC + forage; (23, 24) 1 or 2% blend S^+^ BC; (25, 26) 1 or 2% blend S^+^ BC + forage. 

### 3.5. Seeding and Watering

Four *V. unguiculata* seeds were planted into each pot at 1 cm soil depth. Once seedlings were fully established at 2 weeks, they were thinned down to 2 plants/pot. Because *C. dactylon* propagates vegetatively, it was pre-cultured before the experiment, and a 15 cm sprig was transplanted into each pot. The pots were watered as needed (every 3 to 7 days, depending on temperatures) to near field capacity and the leachate was recycled back into the soil. The experiment was conducted in the greenhouse for 90 days. All treatment combinations were applied in triplicate pots constituting three blocks which consisted of tables within the greenhouse.

### 3.6. Sampling and Sample Preparation

#### 3.6.1. Soil

At trial termination, a soil sample representing 0.5% of total pot soil was taken from each experimental unit (pot) using a small soil probe to minimize root loss and account for a complete cross-section of soil. The samples were allowed to air-dry under ambient conditions until weight stabilized, then sifted.

#### 3.6.2. Forage

The plants were sheared at soil level two times after sowing or planting. After the second cut, the roots were washed with water to remove all remaining soil. All samples were dried in a forced-air oven at 55 °C until weight stabilized. Biomass was recorded immediately after removal from the oven to determine herbage and root DM tissue yields. All samples were ground though a 1171H10 Wiley mill (Thomas Scientific, Swedesboro, NJ, USA) fitted with a 1 mm screen.

### 3.7. Sample Analysis

Soil samples were assayed for the determination of permanganate oxidizable carbon using a method adapted by Culman et al. [28]. Soil samples were additionally assayed for micronutrients by Texas A&M AgriLife Extension Service—Soil, Water, and Forage Testing Laboratory using extractants described by Mehlich [29]. This extract is loosely equivalent to bio-available nutrients since samples are digested in a mildly acidic solution. Data received from this lab included pH, conductivity, P, K, Ca, Mg, S, Na, Fe, Zn, Mn, and Cu values. Additionally, soil NO_3_-N data were assayed using a Cd reduction [30,31]. Total volatilizable N and C percentages were determined by combustion in a Leco CN828 (Leco Corporation, St. Joseph, MI, USA).

Plant samples were assayed for C and N content via CN828 elemental analysis by combustion (LECO Corporation, St. Joseph, MI, USA). For herbage and root samples, the percentages given by the assay were multiplied by the total weight of herbage (two harvests) and root samples to determine the total weight of C and N in grams.

### 3.8. Statistical Analysis

Data were analyzed using R (R-4.2.2) (R Core Team, 2022). Independent variables consisted of BC type, BC saturation, BC loading percentage, and forage inclusion. Dependent variables consisted of soil- and plant-captured N, P, and C percentage, as well as other nutrient and soil health indicators such as dry weight C, N, herbage, and root tissue yields.

Data collected were normally distributed and showed variance homogeneity, so parametric data analyses were used. A Tukey’s test was used to test for differences among dependent variables grouped by treatment. We considered significance at *p* ≤ 0.05 and did not report individual probabilities in text unless they were *p* > 0.05 and relevant to the discussion. Also, Pearson’s correlation analyses and multiple regression analyses (forward) were performed to find associations and cause–effect relationships among plant and soil parameters.

## 4. Discussion

### 4.1. Plant Parameters

The application of saturated or unsaturated BC made from dairy manure at 1% to 2% loading rates negatively affected *V. unguiculata* total herbage production by −18% and −70%, respectively. The first herbage cut showed a 61% and 8% reduction in production and %C but only at the highest loading rate. For the second herbage cut, the manure BC’s negative effect on production (−40%) was observed even at the lowest loading rate, although at this stage, nutritive value was not affected. On the other hand, when applied at the highest loading rate, whichever BC type was applied, root production was negatively affected by −40%, although no impact on nutritive value was observed. However, when applied at the lowest loading rate, no effect on root production or nutrients was observed. The neutral effect of BC application in sandy loam (i.e., 1% wood and blend BC) on *V. unguiculata* growth found in our study was also reported by Taiwo et al. [23] at similar loading rates; however, no information about the BC feedstock was provided in their study. 

Pudasaini et al. [25], in another greenhouse trial with the same species and BC loading rates as the current study and in loamy sandy soil, reported a total plant biomass increase when wood BC was applied. Previously, the same authors had reported a shoot biomass increase in *Capsicum* when 1.6% wood BC was applied in sandy loam soil [32]. Also, Rafael et al. [26] reported greater *V. unguiculata* performance when different types of BC, including wood-based BC, were applied in an acidic arenosol at loading rates lower than 1%. However, in the cited studies, different types and doses of fertilizer were added to the experimental pots. Regarding field experiments, *V. unguiculata* growing in sandy soil showed greater plant growth when wood BC was applied alone even at loading rates < 1% [27]. Also, Southavong et al. [24] reported greater *V. unguiculata* yield when rice husk BC was applied alone. 

In the case of *C. dactylon,* neither herbage and root production nor quality were affected at the end of the trial. However, some partial negative effects were observed for herbage C% (−2%) and production (−27%) at the first and second cut, respectively, independently of the applied BC type and saturation treatment. These results did not agree with Artiola et al. [33] and Niraula et al. [21], who reported an overall increase in the growth of *C. dactylon* when wood BC was applied. Moreover, the application of either saturated wood BC positively affected *C. dactylon* at all assayed loading rates (1 to 8%) and unsaturated wood BC had a positive impact only at the highest loading rate (8%) [21]. In our study, regardless of the BC type, the saturation treatment with dairy effluent did not show a role in any forage performance. 

Other greenhouse experiments examining BC soil amendment application effects on forage grasses and/or legumes reported mixed results. For instance, *Trifolium incarnatum*, a cool-season legume, responded negatively to increasing loading percentages of manure, wood, and blend BC, while *Lolium multiflorum*, a cool-season annual grass, responded positively [22]. However, in our study, the targeted species were not affected (*C. dactylon*) or were only negatively affected (*V. unguiculata*) by manure BC application (herbage biomass) or by the highest loading rate of any BC (root biomass). The neutral effect of all treatments observed for *C. dactylon* performance partially differed from other studies for the same species, which reported a positive effect of saturated (all loading rates) and unsaturated (highest loading rates) wood BC, and a negative (low loading rates) or neutral (intermediate loading rate) effect for the last-mentioned BC treatment [21]. This difference may be because the Niraula et al.’s [21] BC was ground and sieved into <100 µm particles, whereas in our study, BC was only ground through a 2 mm screen. According to Das et al. [34] and Yu and Kuzyakov [35], small particles are more mobile and can have higher reactivity, surface charge, and radical content. Moreover, small particles can also have greater surface area than larger ones [36], which can increase reactivity and nutrient availability [37]. 

### 4.2. Soil Parameters

#### 4.2.1. Macronutrients

For both species, soil total C content increased as BC load increased. This result agreed with the tendency reported by Demisie et al. [38] when wood and bamboo BC were applied in a clay loam soil. In the case of *V. unguiculata*, differences related to the BC type were observed. The highest and lowest soil total C values were registered when wood and manure BC were applied, respectively, while blend BC showed an intermediate value. This may be expected with the fixed carbon content tendency obtained for the studied BCs (see Table 12). 

Soil oxidizable C was not affected by any treatment in any of the studied species. However, in the case of *V. unguiculata*, the application of saturated BC, regardless of the type and loading rate, contributed to maintaining oxidizable soil C content when compared with unsaturated treatment. These results agree with other studies where no changes in oxidizable C content were reported when the same types of BC were tested in *T. incarnatum* and *L. multiflorum* [22]. It is worth noting that saturated BC contributed to retaining soil oxidizable C levels when compared with the unsaturated treatment. This may be because the BC saturation with dairy effluent is expected to increase the initial nutrient loading, although saturation had no discernable effect on plant performance. Biochar surfaces can adsorb nutrients (e.g., N, P) that are abundant in dairy wastewater [39], thereby making them available for a long period of time [40]. 

NO_3_-N, in general, was not affected by any BC treatment; however, regardless of any other factor, 86% and 88% reduction in NO_3_-N resulted when *C. dactylon* or *V. unguiculata*, respectively, were present, likely due to their uptake for growing and because of the conversion to unavailable forms. The soil total N was not affected by any factor or combination in either of the studied species. Unlike *C. dactylon*, when *V. unguiculata* was cultivated, BC application seemed to contribute to some extent to lower soil N losses. This might be because BC can bind forage-available N fixed by legumes. This result did not agree with the reported general tendency that BC treatments increase soil N content [5], nor with other studies that also reported this tendency when saturated or unsaturated wood BC was applied in an experiment carried out with same grass species and soil [21]. 

However, P declined by −18% in the presence of *C. dactylon* but not when *V. unguiculata* was grown. In both studied species, the application of manure or blend BC clearly increased P soil content in the range of 135 to 314%, while wood BC did not increase soil P content. We found that this nutrient was negatively correlated (r = −0.64) with *V. unguiculata* root dry weight, and according to multiple regression analysis, P explained 40% of this negative impact in *V. unguiculata* root performance. However, no positive or negative effect on *C. dactylon* growth was observed. This partially agrees with a study which reported similar BC effect on P soil content despite an overall negative and positive effect on *T. incarnatum* and *L. multiflorum* performance, respectively [22]. Furthermore, our results did not agree with other studies which reported that P content increased when wood BC was applied in an experiment where *C. dactylon* was grown in a sandy loam [21]. Phosphorus binds to BC particles, and its greater presence could be positive in the long term for soil health and plant growth. On the other hand, because BC adsorption ability is linked to P concentration (at high [P], the P-sorption rate slows due to competition for binding sites) [41], excessive P in soils might increase nutrient runoff with negative impacts on downstream ecosystems (i.e., eutrophication). 

In the case of K, *C. dactylon* uptake occurred regardless of the BC loading rate, whereas the presence of *V. unguiculata* showed no differences (exception: 1% blend > 1% blend + *V. unguiculata*). Considering both studied species, independent of whether the plants were present or not, K content increased in the range of 88 to 351% when manure or blend BC was applied. Narwall et al. [42] reported a positive effect on *V. unguiculata* dry matter herbage yield as K loading rates increased, although they studied this effect up to 150 mg/kg loading rates. By contrast, in our study, soil content was around 200 mg/kg and when manure or blend BC were applied, it increased by at least 88%. This may explain, in part, the *V. unguiculata* total herbage weight decrease as well as the root dry weight decrease, since both negatively correlated with soil K content.

The presence of *V. unguiculata* showed a 39% increase in soil Na, whereas for *C. dactylon*, no effect was observed. Considering both species, regardless of the saturation treatment, 2% manure or blend BC increased Na content, although 1–2% blend BC + *C. dactylon* showed a neutral effect. However, when unsaturated, 1% manure BC also increased Na. Taffouo et al. [43] reported that root, stem, and leaf dry weight yields decreased in several *V. unguiculata* cultivars with increasing NaCl concentration. This negative effect was also observed in our study, where Na soil content was negatively correlated with *V. unguiculata* herbage (r = −0.64; Table 10) and root dry weight (r = −0.48; Table 10) yields. Finally, no impact was observed in Ca soil content. *C. dactylon* Ca uptake decreased by −7%, whereas no differences were observed with the inclusion of *V. unguiculata*. For both species, manure BC application resulted in a slightly greater soil Ca content (7–8%), although in the case of *C. dactylon* blend, BC did not differ from manure BC. Other studies on *V. unguiculata* reported an increase [27] or a decrease [23] in soil Ca content when BC was applied in a field and greenhouse trial, respectively. 

Soil Mg increased by 39% when *V. unguiculata* was grown, whereas when *C. dactylon* was cultivated, a neutral effect was observed. For both studied species, the application of manure or blend BC increased Mg soil content. In the case of *V. unguiculata*, as manure BC loading rate increased (1%, 2%), Mg increased by 52 and 87%, respectively. Regardless of the loading rate, the application of manure BC resulted in greater soil Mg compared to the other BC types (manure > blend > wood). The blend BC increased Mg content by 47% only at the highest loading rate. In the case of *C. dactylon*, the manure or blend BC had similar effects when applied at the lowest load, increasing soil Mg content by an average of 34% compared with the control and showing no differences from their respective highest load treatments.

Finally, for both the legume and grass species, the application of manure BC increased soil S content by 32 and 38%, respectively. However, for *C. dactylon*, this effect was observed only at the highest loading rate. An increasing effect of BC on soil Mg content was also detected by Taiwo et al. [23] in a greenhouse experiment with *V. unguiculata*; however, unlike our study, they reported a neutral effect on plant growth. Also, unlike our study, a field trial with this species showed an increase in soil Mg content when wood BC was applied, with a positive effect on plant growth and yield [27]. In our research, the increase in Mg soil content due to the application of any BC may be associated with a negative impact on *V. unguiculata* performance, since it was negatively correlated (r = −0.49) with root dry weight. The increase in soil S content due to manure BC application may have had a negative impact on *V. unguiculata* performance, since it was negatively correlated (r = −0.78, Table 10) with herbage dry weight. 

#### 4.2.2. Micronutrients

Soil Mn decreased by 10% when *C. dactylon* was grown, whereas no decrease was observed for *V. unguiculata*. When *V. unguiculata* and *C. dactylon* were grown, the application of wood BC showed more soil Mn content (16 and 27%, respectively) than the other BC types. For *V. unguiculata*, independently of the BC type, when 2% saturated BC was applied, soil Mn content decreased. 

The inclusion of *V. unguiculata* slightly increased soil Cu and Fe content (23 and 13%, respectively) whereas the application of saturated blend BC decreased them by 24%. For pots growing *C. dactylon,* saturated blend BC reduced only soil Cu content (18%). The application of BC, regardless of the loading rate, decreased soil Fe content by 23 and 27% when *V. unguiculata* and *C. dactylon* were grown, respectively. No effect in Zn content was observed when *C. dactylon* was included, whereas it was reduced by 8% due to *V. unguiculata* uptake. When *V. unguiculata* was included, saturated BC reduced soil Fe by 7% when compared with unsaturated BC; however, both treatments showed values close to control soil content. In our study, soil metal contents were neither limiting nor toxic, since no negative correlation with biomass production was detected. However, we detected that the application of BC decreased soil Fe content when both species were grown and Cu soil content when *V. unguiculata* was grown. This might be because cations in soils such as Fe^3+^ can be immobilized by BC in an anion/cation exchange and held there as a possible adsorption site for anions [41,44]. Regarding Cu and Zn, besides the effect of plant uptake, soil-incorporated BC can stabilize them and reduce their bioavailability through enhanced sorption and chemical precipitation [45]. In that way, as the water-soluble, bioactive fraction of heavy metals in soil decreases, potential uptake and bioaccumulation of heavy metals by soil organisms (including plant roots) are minimized [46]. In our study, this could be desirable for Cu, since soil content (0.3 mg/kg) is higher than the critical value (0.1 mg/kg) reported for *V. unguiculata* [47] but undesirable in the case of Zn, where concentration in the soil (0.8 mg/kg) is slightly under the critical level (1 mg/kg) [48]. 

#### 4.2.3. pH and Conductivity 

In pots growing *V. unguiculata,* manure and blend BC increased soil pH as loading rate increased, while wood BC increased it only at the highest assayed loading rate. At the highest BC loading rate, all treatments had differing positive effects on soil pH [manure BC (9%) > blend BC (5%) > wood BC (2.5%)]. In the case of *C. dactylon,* the application of 1 or 2% manure and 2% blend BC increased pH. Because there was only a pH 0.6 difference between the control pH (7.71) and the highest values (8.32 and 8.34) corresponding to 2% manure BC + *V. unguiculata* or *C. dactylon*, respectively, it may be that this difference is too slight to have an impact in soil and plant parameters. However, according to regression analysis, pH increases mainly due to conductivity increase (R^2^ = 0.79). This last parameter increase was negatively correlated with *V. unguiculata* growth. For both species, only the manure BC treatment differed from the control with increasing conductivity. *Vigna unguiculata* increased soil conductivity by 5 and 106% at the 1% and 2% BC loading rates, respectively, whereas *C. dactylon* increased by 71% when 2% manure BC was applied. Conductivity was negatively correlated with *V. unguiculata* total herbage weight and multiple regression analysis showed that this soil parameter explained the negative impact on *V. unguiculata* performance (i.e., total herbage dry weight) (R^2^ = 0.74). Excess salts or high conductivity in soil is harmful for plants, because of a decrease in osmotic potential; therefore, the conductivity of the soil must be kept low for desirable nutrient availability and plant growth [49]. This is a challenge, because most BCs contain high amounts of soluble salts, and hence, their conductivity is generally higher than most agricultural soils [50]. 

## 5. Conclusions

The first notable conclusion is that the application of high BC rates, whether saturated or not, is detrimental in the short term for *V. unguiculata* performance but shows a neutral effect for *C. dactylon*. In that sense, all BC types when applied at the highest loading rate negatively affected *V. unguiculata* root biomass, whereas only manure BC, regardless of the loading rate, affected the herbage biomass. This may be interpreted in a positive sense. If the objective is to reuse dairy production wastes to fertilize dairy forage production in an effort to close the farm nutrient cycle, this could be accomplished using 1% blend BC to avoid negative effects on *V. unguiculata* performance. For *C. dactylon*, it would be possible, if economically and biologically desirable, to apply greater loads of manure or blend BC. This would reuse dairy-concentrated animal feeding operation wastes as well as increase some soil nutrient levels without short-term negative impacts on forage production performance. However, it is worth noting that manure and blend BC increased soil P levels, while wood BC did not. Because incorporating BC improves soil total carbon content, BC is an appropriate alternative to consider among the strategies for long-term carbon sequestration. 

The differences between the studied grass and legume responses need further elucidation. We hypothesized that the fibrous nature of grass roots may facilitate nutrient uptake vis-à-vis the less fibrous legume taproot system when BC binds some soil nutrients, making them unavailable for plant development. The more extensive grass root system may facilitate contact with the fewer remaining soil nutrients. This would be relevant especially for short-lived annual forages. 

Saturating BC with dairy effluent, because it is useful to reduce liquid dairy waste nutrient loads and resulting environmental contamination (e.g., via runoff and eutrophication), could be a viable alternative due to having no negative impact in plant performance. Although it made no difference when *C. dactylon* was grown, in the case of *V. unguiculata*, saturated BC increased soil oxidizable C. 

## Figures and Tables

**Table 1:** *Vigna unguiculata* first herbage cut production (g H-DW1/pot) and quality (H-DW1C%) and *Cynodon dactylon* quality (H-DW1C%) with Tukey’s test displaying the mean ± standard error; (a) ANOVA two-way interaction: biochar (BC) type × BC loading rate (*p* < 0.001); (b) ANOVA one-way interaction: BC loading (%) (*p* = 0.045).

*Vigna unguiculata*				
(a)				
		BC loading (%)		
Plant parameter	BC type	0	1	2
	Manure	18.6 ± 2.19 a A *	18.15 ± 2.58 b A	6.75 ± 1.27 c B
H-DW1 (g/pot)	Blend	13.6 ± 1.61 a A	18.80 ± 3.00 a A	18.60 ± 2.40 a A
	Wood	11.1 ± 1.46 a A	20.63 ± 1.70 a A	20.21 ± 1.82 a A
	Manure	41.6 ± 0.21 a A	40.03 ± 0.38 a A	37.96 ± 0.40 b B
H-DW1 (C%)	Blend	40.7 ± 0.24 a A	39.68 ± 0.55 a A	39.85 ± 0.32 a A
	Wood	41.2 ± 0.48 a A	40.90 ± 0.30 a A	
*Cynodon dactylon*				
(b)				
		BC loading (%)		
		0	1	2
H-DW1 (C%)		41.33 ± 0.15 a *	40.72 ± 0.19 ab	40.50 ± 0.29 b

* Within each species and plant parameter, values within each column (upper case) and each line (lower case) followed by the same letter do not differ (*p* ≤ 0.05) according to a least significant multiple mean separation.

**Table 2:** *Vigna unguiculata* and *Cynodon dactylon* second herbage cut production (g H-DW2/pot) as affected by biochar (BC) type, BC loading rate, and BC saturation with Tukey’s test displaying the mean ± standard error; ANOVA two-way interaction for *V. unguiculata*: (a) BC type × BC loading (*p* < 0.001); (b) BC type × BC saturation (*p* = 0.015); ANOVA one-way interaction for *C. dactylon*: BC loading (%) (*p* = 0.043).

*Vigna unguiculata*				
(a)				
		BC loading (%)		
		0	1	2
	Manure	12.5 ± 2.12 a A *	4.81 ± 0.76 b A	1.21 ± 0.31 b A
BC type	Blend	6.5 ± 1.25 a A	9.06 ± 1.62 a A	7.25 ± 0.70 a A
	Wood	5.1 ± 0.76 a A	20.63 ± 0.75 a A	20.21 ± 1.23 a A
(b)				
		BC saturation		
		NO	YES	
	Manure	4.48 ± 1.30 a B *	7.86 ± 2.30 a A	
BC type	Blend	8.52 ± 1.24 a A	6.67 ± 0.70 a A	
	Wood	6.34 ± 1.01 a AB	7.31 ± 0.75 a A	
		*Cynodon dactylon*		
		BC loading (%)		
Plant Parameter		0	1	2
H-DW2 (g/pot)		15.85 ± 1.15 a *	11.52 ± 1.44 b	13.25 ± 0.75 ab

* Within each species, interaction, and plant parameter, values within each column (upper case) and each line (lower case) followed by the same letter do not differ (*p* ≤ 0.05) according to a least significant multiple mean separation.

**Table 3:** *Vigna unguiculata* total herbage production (g TH-DW/pot) with Tukey’s test displaying the mean ± standard error; ANOVA two-way interaction: biochar (BC) type × BC loading rate (*p* = 0.01).

BC Type	BC Loading (%)		
	0	1	2
Manure	30.5 ± 4.20 a A *	18.15 ± 2.20 b A	6.75 ± 1.39 c B
Blend	20.1 ± 2.61 a A	18.80 ± 3.47 a A	18.60 ± 2.70 a A
Wood	19.7 ± 2.40 a A	20.60 ± 2.04 a A	20.20 ± 2.60 a A

* Values within each column (upper case) and each line (lower case) followed by the same letter do not differ (*p* ≤ 0.05) according to a least significant multiple mean separation.

**Table 4:** *Vigna unguiculata* root production (g R-DW/pot) with Tukey’s test displaying the mean ± standard error; ANOVA one-way interaction: biochar (BC) loading (%) (*p* = 0.024). Values followed by the same letter do not differ (*p* ≤ 0.05) according to a least significant multiple mean separation.

BC Loading (%)	Root Dry Weight (g/pot)
0	11.76 ± 1.45 a
1	9.48 ± 0.76 ab
2	6.95 ± 0.63 b

**Table 5:** Soil total and oxidizable C, nitrates (NO_3_-N), P, potassium (K), sodium (Na), and calcium (Ca) alteration with Tukey’s test displaying the mean ± standard error for *Vigna unguiculata* and *Cynodon dactylon*; ANOVA one-way interaction. Different letters within each nutrient and factor indicate significant differences (*p* ≤ 0.05) according to a least significant multiple mean separation.

	Soil Macronutrient								
	g/kg				mg/kg				
Factor	Total C	Oxidizable C	NO_3_-N	P	K	Na	Ca	Mg	S
*Vigna unguiculata*									
Plant application									
NO	34.3 ± 0.10 a	209.0 ± 21.5 a	10.6 ± 0.95 a	62.56 ± 5.62 a	335.8 ± 27.6 a	70.2 ± 4.78 b	1671 ± 35.2 a	211.3 ± 8.52 b	26.1 ± 1.57 a
YES	34.8 ± 0.11 a	230.7 ± 21.7 a	1.3 ± 0.10 b	58.49 ± 6.33 a	229.7 ± 27.3 a	97.3 ± 6.02 a	1597 ± 30.9 a	246.2 ± 9.44 a	23.5 ± 1.53 a
*p*-value			<0.001			<0.001		<0.001	
Biochar type									
Manure	32.4 ± 0.11 b	200.1 ± 19.0 a	3.94 ± 1.07 a	85.64 ± 8.94 a	397.9 ± 45.3 a	100.7 ± 9.1 a	1742 ± 32.0 a	275.0 ± 12.68 a	26.7 ± 2.03 a
Blend	34.4 ± 0.14 ab	226.1 ± 28.6 a	5.97 ± 1.13 a	63.84 ± 5.55 a	272.1 ± 21.9 a	83.5 ± 5.1 a	1599 ± 35.8 b	229.7 ± 9.02 a	25.4 ± 1.80 a
Wood	36.9 ± 0.15 a	236.5 ± 30.5 a	6.72 ± 1.21 a	29.25 ± 1.34 a	152.1 ± 8.9 a	60.2 ± 3.0 a	1569 ± 47.7 b	181.6 ± 5.18 a	22.4 ± 1.86 a
*p*-value	0.027						0.001		
Biochar loading (%)									
0	28.8 ± 0.08 c	258.9 ± 41.8 a	4.07 ± 1.07 a	29.00 ± 2.7 a	147.6 ± 15.3 a	56.8 ± 5.21 a	1590.2 ± 43.7 a	191.9 ± 8.77 a	17.6 ± 1.90 a
1	35.0 ± 0.11 b	164.8 ± 25.6 a	5.32 ± 2.03 a	91.12 ± 7.4 a	389.5 ± 32.5 a	108.3 ± 12.0 a	1753.2 ± 46.8 a	283.9 ± 13.45 a	26.4 ± 9.91 a
2	39.9 ± 0.13 a	198.8 ± 29.2 a	6.23 ± 2.33 a	145.30 ± 9.2 a	735.2 ± 49.8 a	157.4 ± 12.4 a	1884.5 ± 41.9 a	349.1 ± 11.95 a	36.1 ± 3.41 a
*p*-value	<0.001								
Biochar saturation									
NO	34.5 ± 0.11 a	181.1 ± 12.88 b	7.2 ± 1.12 a	66.5 ± 6.62 a	300.2 ± 31.58 a	87.15 ± 6.47 a	1644.7 ± 35.0 a	233.8 ± 10.05 a	26.7 ± 1.78 a
YES	34.6 ± 0.11 a	258.6 ± 26.81 a	4.7 ± 0.63 b	54.5 ± 5.16 b	265.3 ± 24.64 b	80.34 ± 4.87 a	1623.9 ± 32.0 a	223.8 ± 8.44 a	22.9 ± 1.25 a
*p*-value		0.038	0.007	0.002	0.035				
*Cynodon dactylon*									
Plant application									
NO	34.30 ± 0.10 a	209.0 ± 21.54 a	10.60 ± 0.95 a	62.5 ± 5.62 a	335.8 ± 27.64 a	70.20 ± 4.78 a	1671.2 ± 35.3 a	211.3 ± 8.52 a	26.11 ± 1.57 a
YES	34.2 ± 0.13 a	202.5 ± 16.47 a	1.50 ± 0.18 b	51.4 ± 4.79 b	248.1 ± 24.83 a	74.51 ± 4.91 a	1567.4 ± 27.8 b	215.6 ± 7.41 a	29.47 ± 1.66 a
*p*-value			<0.001	<0.001			0.035		
BC type									
Manure	32.10 ± 0.09 a	197.3 ± 22.92 a	5.27 ± 1.07 a	82.33 ± 7.77 a	425.0 ± 42.97 a	92.56 ± 7.47 a	1699.0 ± 34.0 a	255.5 ± 11.20 a	29.40 ± 1.89 a
Blend	35.70 ± 0.14 a	192.6 ± 23.89 a	6.23 ± 1.11 a	58.60 ± 5.08 a	286.9 ± 20.80 a	74.01 ± 4.48 a	586.6 ± 35.1 ab	212.7 ± 7.11 a	27.90 ± 2.13 a
Wood	35.00 ± 0.18 a	185.7 ± 26.16 a	6.67 ± 1.22 a	30.13 ± 1.15 a	164.0 ± 7.04 a	50.48 ± 2.40 a	1572.2 ± 46.4 b	172.3 ± 3.96 a	26.08 ± 1.95 a
*p*-value							0.039		
BC loading (%)									
0	28.10 ± 0.07 c	219.2 ± 27.46 a	5.87 ± 1.00 a	30.87 ± 1.46 a	156.6 ± 8.02 a	49.05 ± 2.00 a	1581.7 ± 41.64 a	177.5 ± 3.61 a	24.33 ± 1.69 b
1	34.90 ± 0.10 b	185.4 ± 22.09 a	5.87 ± 0.99 a	61.80 ± 4.57 a	292.8 ± 20.85 a	71.80 ± 3.99 a	1618.7 ± 32.78 a	218.3 ± 7.79 a	26.83 ± 1.63 ab
2	39.80 ± 0.17 a	171.0 ± 22.47 a	6.48 ± 1.40 a	78.38 ± 8.40 a	426.5 ± 41.97 a	96.21 ± 7.44 a	1657.4 ± 43.85 a	244.6 ± 12.30 a	32.21 ± 2.37 a
*p*-value	<0.001								0.022

**Table 6:** Soil P, potassium (K), sodium (Na), magnesium (Mg), and sulfur (S) as affected by biochar (BC) type, BC loading rate, BC saturation, and plant application with Tukey’s test displaying the mean ± standard error for *Vigna unguiculata* and *Cynodon dactylon*. ANOVA three- and two-way interactions (*p* ≤ 0.05) for *V. unguiculata*: (a) BC type × BC loading rate × plant application; (b) BC type × BC loading rate × BC saturation; (c) BC type × BC loading rate; (d) BC saturation × plant application. ANOVA three- and two-way interactions (*p* ≤ 0.05) for *C. dactylon*: (a) BC type × BC loading rate × BC saturation; (b) BC type × BC loading rate; (c) BC loading rate × plant application; (d) BC saturation × plant application.

*Vigna unguiculata*				
(a)				
Nutrient (*p*-value)	BC type (Plant application)	BC loading (%)		
		0	1	2
P (mg/kg) (*p =* 0.025)	Manure (NO)	35.2 ± 3.07 b AB *	93.3 ± 8.54 a A	145.5 ± 13.91 a A
	Manure (YES)	22.7 ± 2.63 b B	88.9 ± 12.81a A	145.1 ± 13.64 a A
	Blend (NO)	35.2 ± 4.79 b AB	65.9 ± 6.44 a A	87.3 ± 12.09 a B
	Blend (YES)	21.8 ± 1.62 c B	58.9 ± 4.63 b AB	109.1 ± 8.29 a AB
	Wood (NO)	35.2 ± 2.26 a AB	36.5 ± 1.78 a B	28.5 ± 2.49 a C
	Wood (YES)	28.5 ± 5.17 a AB	28.5 ± 3.39 a B	22.6 ± 1.59 a C
K (mg/kg) (*p* = 0.025)	Manure (NO)	199.3 ± 8.88 c A	456.5 ± 41.94 b A	776.1 ± 75.36 a A
	Manure (YES)	100.5 ± 7.93 c B	322.4 ± 32.99 b A	694.1 ± 67.64 a A
	Blend (NO)	199.3 ± 10.07 b A	328.7 ± 29.42 a A	454.8 ± 39.48 a B
	Blend (YES)	95.7 ± 6.66 c B	203.8 ± 13.96 b B	340.3 ± 33.86 a B
	Wood (NO)	199.3 ± 8.17 a A	207.6 ± 3.88 a B	200.4 ± 10.59 a C
	Wood (YES)	109.6 ± 6.34 a B	101.2 ± 4.05 a C	100.0 ± 8.12 a D
(b)				
	BC type (BC saturation)	BC loading (%)		
		0	1	2
Na (mg/kg) (*p* = 0.021)	Manure (NO)	53.6 ± 5.48 b A *	131.5 ± 17.25 a A	170.7 ± 19.72 a A
	Manure (YES)	59.8 ± 9.23 b A	85.1 ± 10.89 b AB	144.2 ± 14.78 a AB
	Blend (NO)	52.9 ± 5.28 b A	83.8 ± 7.86 ab AB	116.3 ± 12.31 a AB
	Blend (YES)	60.6 ± 6.74 b A	81.9 ± 10.34 ab AB	105.3 ± 11.10 a ABC
	Wood (NO)	60.2 ± 4.50 a A	56.3 ± 4.97 a B	58.6 ± 8.85 a C
	Wood (YES)	48.7 ± 7.27 a A	67.0 ± 4.70 a B	70.1 ± 11.40 a C
(c)				
		BC loading (%)		
	BC type	0	1	2
Mg (mg/kg) (*p* < 0.001)	Manure	191.9 ± 8.77 c A *	283.9 ± 13.45 b A	349.1 ± 11.95 a A
	Blend	187.2 ± 7.75 b A	227.6 ± 10.01 ab B	274.4 ± 16.46 a B
	Wood	181.4 ± 9.21 b A	183.8 ± 9.19 a C	179.6 ± 9.27 a C
S (mg/kg) (*p* = 0.035)	Manure	17.5 ± 1.90 b	26.3 ± 2.90 a A	30.1 ± 3.41 a A
	Blend	21.3 ± 3.06 a	36.5 ± 1.78 a A	31.3 ± 3.64 a A
	Wood	22.1 ± 2.20 a	28.5 ± 3.20 a A	24.6 ± 4.13 a A
(d)				
Plant application				
	BC saturation	NO	YES	
S (mg/kg)	NO	29.5 ± 2.35 b A *	23.9 ± 2.61 a A	
(*p* = 0.04)	YES	22.7 ± 1.91 a A	23.1 ± 1.64 a A	
*Cynodon dactylon*				
(a)				
	BC type (BC saturation)	BC loading (%)		
		0	1	2
Na (mg/kg) (*p* = 0.035)	Manure (NO)	46.7 ± 3.58 b A *	102.8 ± 6.57 a A	146.4 ± 18.39 a A
	Manure (YES)	51.1 ± 6.69 b B	79.0 ± 8.87 ab AB	129.3 ± 12.34 a A
	Blend (NO)	49.1 ± 4.03 b A	79.8 ± 7.05 ab AB	116.3 ± 12.49 a AB
	Blend (YES)	55.8 ± 5.90 a A	68.0 ± 7.75 a ABC	105.3 ± 11.75 a ABC
	Wood (NO)	51.9 ± 3.19 a A	44.1 ± 1.92 a C	58.6 ± 6.43 a C
	Wood (YES)	39.6 ± 4.36 a A	57.0 ± 4.34 a BC	60.2 ± 9.51 a B
(b)				
		BC loading (%)		
	BC type	0	1	2
P (mg/kg) (*p* < 0.001)	Manure	31.9 ± 2.13 c A *	87.2 ± 5.86 b A	127.7 ± 10.88 a A
	Blend	30.5 ± 3.70 b A	63.7 ± 5.77 a A	81.5 ± 8.83 a B
	Wood	30.1 ± 1.44 a A	34.4 ± 2.07 a B	25.8 ± 1.72 a C
K (mg/kg) (*p* < 0.001)	Manure	151.1 ± 14.36 c A	417.4 ± 24.89 b A	706.3 ± 52.75 a A
	Blend	163.4 ± 15.37 c A	294.2 ± 22.74 b B	402.8 ± 27.67 a B
	Wood	155.1 ± 12.83 a A	166.7 ± 12.88 a C	170.3 ± 11.49 a C
Mg (mg/kg) (*p* < 0.001)	Manure	183.8 ± 5.41 b A	264.1 ± 8.66 a A	318.4 ± 16.05 a A
	Blend	177.0 ± 5.21 b A	220.0 ± 8.71 a A	240.8 ± 13.67 a B
	Wood	171.5 ± 7.82 a A	170.8 ± 5.68 a B	174.5 ± 7.46 a C
(c)				
		Plant application		
	BC loading (%)	NO	YES	
K (mg/kg)	0	199.3 ± 5.21 a C *	113.8 ± 4.75 b C	
(*p* = 0.002)	1	330.9 ± 29.42 a B	254.6 ± 27.45 b B	
	2	477.1 ± 63.12 a A	375.8 ± 54.48 b A	
(d)				
Plant application				
	BC saturation	NO	YES	
S (mg/kg)	NO	29.5 ± 2.35 b A *	28.3 ± 2.10 a A	
(*p* = 0.039)	YES	22.7 ± 1.91 a A	30.6 ± 2.59 a A	

* Within each species, interaction, and nutrient, values within each column (upper case) and each line (lower case) followed by the same letter do not differ (*p* ≤ 0.05) according to a least significant multiple mean separation.

**Table 7:** Soil manganese (Mn), copper (Cu), iron (Fe), and zinc (Zn) as affected by biochar (BC) type, BC loading rate, BC saturation, and plant application with Tukey’s test displaying the mean ± standard error for *Vigna unguiculata* and *Cynodon dactylon*; ANOVA one-way interaction. Different letters within each nutrient and factor indicate significant differences (*p* ≤ 0.05) according to a least significant multiple mean separation.

	Soil Micronutrient			
			mg/kg	
Factor	Mn	Cu	Fe	Zn
*Vigna unguiculata*				
Plant application				
NO	4.28 ± 0.31 a	0.31 ± 0.09 b	3.12 ± 0.01 b	0.89 ± 0.01 b
YES	8.05 ± 0.42 a	0.38 ± 0.12 a	3.54 ± 0.02 a	0.96 ± 0.01 a
*p*-value		<0.001	0.007	0.032
BC Type				
Manure	5.99 ± 0.58 b	0.35 ± 0.02 a	3.35 ± 0.14 a	0.96 ± 0.03 a
Blend	5.72 ± 0.44 b	0.32 ± 0.02 a	3.12 ± 0.15 a	0.92 ± 0.03 a
Wood	6.77 ± 0.42 a	0.36 ± 0.01 a	3. 53 ± 0.12 a	0.90 ± 0.02 a
*p*-value	0.006			
BC loading (%)				
0	7.60 ± 1.55 a	0.37 ± 0.02 a	3.96 ± 0.31 a	0.87 ± 0.05 a
1	5.83 ± 0.63 a	0.36 ± 0.04 a	3.07 ± 0.16 b	0.94 ± 0.04 a
2	4.57 ± 0.27 a	0.33 ± 0.03 a	3.00 ± 0.13 b	1.07 ± 0.04 a
*p*-value			<0.001	
BC saturation				
NO	5.94 ± 0.30 a	0.35 ± 0.01 a	3.50 ± 0.12 a	0.96 ± 0.02 a
YES	6.40 ± 0.48 a	0.33 ± 0.01 a	3.17 ± 0.10 a	0.89 ± 0.02 b
*p*-value				0.032
*Cynodon dactylon*				
Plant application				
NO	4.28 ± 0.01 a	0.31 ± 0.01 a	3.84 ± 0.09 a	0.89 ± 0.01 a
YES	3.84 ± 0.10 b	0.32 ± 0.01 a	3.12 ± 0.12 b	0.87 ± 0.02 a
*p*-value	<0.001		<0.001	
BC loading (%)				
0	3.82 ± 0.11 a	0.36 ± 0.03 a	4.06 ± 0.16 a	0.86 ± 0.02 a
1	4.05 ± 0.12 a	0.30 ± 0.01 a	3.18 ± 0.10 b	0.82 ± 0.02 a
2	4.30 ± 0.14 a	0.32 ± 0.02 a	3.20 ± 0.10 b	0.96 ± 0.04 a
*p*-value			<0.001	

**Table 8:** Soil manganese (Mn), copper (Cu), and iron (Fe) as affected by biochar (BC) type, BC loading rate, BC saturation with Tukey’s test displaying the mean ± standard error for *Vigna unguiculata* and *Cynodon dactylon*. ANOVA two-way interaction (*p* ≤ 0.05) for *V. unguiculata*: BC type × BC loading rate. ANOVA two-way interactions (*p* ≤ 0.05) for *C. dactylon*: (a) BC type × BC saturation; (b) BC type × BC loading rate.

*Vigna unguiculata*				
		BC saturation		
Nutrient				
(*p*-value)	BC type	NO		YES
Fe (mg/kg)	Manure	3.36 ± 0.21 a A *		3.33 ± 0.19 a A
(*p* = 0.007)	Blend	3.57 ± 0.20 a A		2.66 ± 0.16 b B
	Wood	3.55 ± 0.12 a A		3.51 ± 0.20 a A
Cu (mg/kg)	Manure	0.32 ± 0.02 a A		0.38 ± 0.03 a A
(*p* = 0.005)	Blend	0.37 ± 0.03 a A		0.27 ± 0.01 b B
	Wood	0.37 ± 0.01 a A		0.35 ± 0.02 a A
*Cynodon dactylon*				
(a)				
Mn (mg/kg)	Manure	3.87 ± 0.08 a A *		3.84 ± 0.13 a A
(*p* = 0.033)	Blend	4.22 ± 0.16 a A		3.58 ± 0.19 b B
	Wood	4.38 ± 0.20 a A		4.45 ± 0.20 a A
Fe (mg/kg)	Manure	3.36 ± 0.17 a A		3.61 ± 0.20 a A
(*p* = 0.005)	Blend	3.59 ± 0.20 a A		2.90 ± 0.20 b B
	Wood	3.68 ± 0.16 a A		3.74 ± 0.21 a A
Cu (mg/kg)	Manure	0.303 ± 0.01 a A		0.345 ± 0.02 a A
(*p* = 0.002)	Blend	0.332 ± 0.02 a A		0.263 ± 0.02 b B
	Wood	0.333 ± 0.05 a A		0.329 ± 0.01 a AB
(b)				
		BC loading (%)		
		0	1	2
Mn (mg/kg)	Manure	3.86 ± 0.11 a A *	3.83 ± 0.14 b A	3.88 ± 0.15 a B
(*p* = 0.008)	Blend	3.93 ± 0.22 a A	3.79 ± 0.26 a A	4.00 ± 0.25 a B
	Wood	3.72 ± 0.24 b A	4.5 ± 0.14 ab A	5.01 ± 0.19 a A

* Within each species, interaction, and nutrient, values within each column (upper case) and each line (lower case) followed by the same letter do not differ (*p* ≤ 0.05) according to a least significant multiple mean separation.

**Table 9:** pH and conductivity as affected by biochar (BC) type, BC loading rate, BC saturation, and plant application with Tukey’s test displaying the mean ± standard error for *Vigna unguiculata* and *Cynodon dactylon*. ANOVA two-way interactions (*p* ≤ 0.05) for *V. unguiculata*: (a) BC type × plant application; (b) BC type × BC saturation; (c) BC type × BC loading rate; (d) BC loading rate × plant application. ANOVA two- and one-way interactions (*p* ≤ 0.05) for *C. dactylon*: (a) BC type × BC loading rate; (b) BC type × BC saturation; (c) plant application.

*Vigna unguiculata*				
(a)				
		Plant application		
Parameter				
(*p*-value)	BC type	NO	YES	
pH (*p* = 0.036)	Manure	8.05 ± 0.06 a A *	7.87 ± 0.08 b A	
	Blend	7.85 ± 0.06 a B	7.83 ± 0.05 a A	
	Wood	7.86 ± 0.03 a B	7.70 ± 0.03 b B	
(b)				
BC saturation				
	BC type	NO	YES	
pH (*p* = 0.005)	Manure	8.03 ± 0.07 a A *	7.89 ± 0.08 a A	
	Blend	7.82 ± 0.06 a A	7.87 ± 0.05 a B	
	Wood	7.82 ± 0.02 a A	7.73 ± 0.04 a B	
(c)				
		BC loading (%)		
	BC type	0	1	2
pH (*p* < 0.001)	Manure	7.62 ± 0.06 c A *	7.96 ± 0.04 b A	8.32 ± 0.04 a A
	Blend	7.64 ± 0.04 c A	7.83 ± 0.08 b AB	8.06 ± 0.02 a B
	Wood	7.63 ± 0.05 b A	7.76 ± 0.03 b B	7.85 ± 0.03 a C
Conductivity (mS/cm) (*p* = 0.035)	Manure	0.15 ± 0.01 c A	0.17 ± 0.01 b A	0.35 ± 0.03 a A
	Blend	0.18 ± 0.01 a A	0.16 ± 0.01 a A	0.25 ± 0.03 a B
	Wood	0.16 ± 0.005 a A	0.14 ± 0.05 a A	0.15 ± 0.09 a C
(d)				
		Plant application		
	BC loading (%)	NO	YES	
Conductivity (mS/cm) (*p* =0.008)	0	0.18 ± 0.01 a A *	0.15 ± 0.008 a B	
	1	0.16 ± 0.09 a A	0.16 ± 0.008 a B	
	2	0.22 ± 0.02 a A	0.28 ± 0.03 a A	
*Cynodon dactylon*				
(a)				
BC loading (%)				
	BC type	0	1	2
pH	Manure	7.73 ± 0.02 c A *	8.03 ± 0.03 b A	8.34 ± 0.04 a A
(*p* = 0.001)	Blend	7.74 ± 0.03 b A	7.81 ± 0.01 b AB	8.08 ± 0.03 a B
	Wood	7.80 ± 0.03 a A	7.86 ± 0.03 a B	7.95 ± 0.02 a B
Conductivity	Manure	156.8 ± 9.72 b A	163.0 ± 10.28 b A	287.1 ± 28.66 a A
(mS/cm)	Blend	184.7 ± 13.69 a A	170.4 ± 11.28 a A	212.3 ± 31.61 a B
(*p* = 0.001)	Wood	161.0 ± 7.44 a A	136.0 ± 5.93 a A	152.9 ± 9.74 a B
(b)				
BC saturation				
	BC type	NO	YES	
pH	Manure	8.12 ± 0.06 a A *	7.98 ± 0.06 a A	
(*p* = 0.01)	Blend	7.83 ± 0.08 a B	7.92 ± 0.03 a A	
	Wood	7.91 ± 0.01 a B	7.84 ± 0.03 a A	
(c)				
		Plant application		
		NO	YES	
Conductivity (mS/cm)		0.19 ± 0.01 a *	0.17 ± 0.08 b	
(*p* = 0.04)				

* Within each species, interaction, and soil parameter, values within each column (upper case) and each line (lower case) followed by the same letter do not differ (*p* ≤ 0.05) according to a least significant multiple mean separation.

**Table 10:** Pearson’s correlation coefficients (*p* ≤ 0.05) for *Vigna unguiculata* when (a) manure biochar was applied (n = 12); (b) 2% biochar was applied (n = 18).

(a)			(b)
	TH-DW	Conductivity	R-DW
oxidizable C	−0.53 ns	0.64 *	−0.40 ns
NO_3_-N	0.04 ns	−0.12 ns	−0.19 ns
P	−0.56 ns	0.77 *	−0.64 *
K	−0.83 *	0.88 *	−0.63 *
Na	−0.64 *	0.71 *	−0.48 *
Ca	−0.17 ns	0.44 ns	−0.27 ns
Mg	−0.38 ns	0.67 *	−0.49 *
Mn	−0.81 *	0.69 *	0.65 *
S	−0.78 *	0.77 *	0.32 ns
Cu	−0.03 ns	0.13 ns	0.35 ns
Fe	0.04 ns	0.04 ns	0.35 ns
Zn	−0.53 ns	0.67 *	−0.21 ns
pH	−0.84 *	0.89 *	−0.57 *
Conductivity	−0.86 *	−−	−0.36 ns

*: significant correlations; ns: not significant correlations; TH-DW: Total herbage dry weight per pot; R-DW: root dry weight per pot.

**Table 11:** Soil characteristic averages.

Chemical Characteristics	Sandy Loam
pH	7.85
Conductivity (mS/cm)	0.166
Oxidizable C (mg/kg)	198.68
NO_3_-N (mg/kg)	10.86
P (mg/kg)	34.77
K (mg/kg)	198.18
Ca (mg/kg)	1820.53
Na (mg/kg)	47.04
Mg (mg/kg)	176.04
S (mg/kg)	26.38
Fe (mg/kg)	3.44
Zn (mg/kg)	0.89
Mn (mg/kg)	4.31
Cu (mg/kg)	0.36
Organic matter	N/A
Total C (g/kg)	32.3
Total N (g/kg)	31.0

**Table 12:** Initial biochar (BC) characterization.

	Wood BC	Blend BC	Manure BC
	%		
Nitrogen	0.211	0.290	0.738
Phosphorus	0.004	0.631	1.149
Potassium	0.214	1.767	4.392
Calcium	0.216	3.649	6.389
Magnesium	0.035	0.722	2.615
Sodium	0.059	0.326	0.742
Ash	5.83	22.94	40.05
Fixed Carbon	60.70	42.27	23.83
Volatile Matter	27.84	30.21	32.57
	mg/kg		
Zinc	36.61	150.18	285.93
Iron	775.36	3721.51	7708.70
Copper	12.62	62.29	153.70
Manganese	139.14	330.85	432.47
Sulfur	13.70	3943.97	3167.22
Boron	2.32	6.22	29.74
pH	8.8	9.4	10.2

## Data Availability

Data are contained within the article.
